# Palm Cooling’s Heart Rate and Thermal Impact on Exercise in Multiple Sclerosis Patients

**DOI:** 10.1055/a-2877-8927

**Published:** 2026-06-12

**Authors:** Katherine Frances Maguire, Margaret Wydotis, Jason Jaggers, Pete Quesada, Jennifer Daily, Yvette Cabrera-Rojas, John Francis Caruso

**Affiliations:** 1 Health & Sport Sciences 5170 University of LouisvilleLouisvilleKentuckyUnited States; 2 Mechanical EngineeringUniversity of LouisvilleLouisvilleKentuckyUnited States; 3 Family & Geriatric Medicine 12254 University of Louisville School of MedicineLouisvilleKentuckyUnited States; 4 Neurology 220113 Norton Neuroscience InstituteLouisvilleKentuckyUnited States

**Keywords:** conduction, cold-induced vasodilation, anastomoses, retia venosa, evaporation

## Abstract

Heat strain in multiple sclerosis patients during exercise limits their benefits from this treatment. One prior study examined this topic but did not measure thermal outcomes and constitutes a knowledge gap. The current pilot study assessed palm cooling for its impact on heart rates and thermal measures. Using a randomized within-subjects design, nine multiple sclerosis patients performed two 25-minute cycle ergometry workouts against 65 and 45% of their Astrand-Rhyming-based workloads. Heart rates, thermal flux, palm and tympanic temperatures were examined using two-way analyses of variance with repeated measures per independent variable. To control the family-wise error rate, a Bonferroni correction was applied at a lower α level of 0.0024. Paired *t* -tests identified sources of significant inter-treatment and -time differences. Palm temperature, thermal flux, and heart rate had significant two-way interactions. Palm temperature ( *η* ^2^_*p*_ =0.39) and heart rate ( *η* ^2^_*p*_ =0.22) results were significantly higher for the no palm cooling treatment at multiple times during and after workouts. Yet, thermal flux ( *η* ^2^_*p*_ =0.39) was significantly higher due to palm cooling at multiple times during and after workouts. Higher thermal flux from palm cooling may have evoked lower heart rates. Palm cooling may represent a practical strategy to mitigate exercise-induced heat strain in individuals with multiple sclerosis.

## Introduction

Physicians recommend exercise to patients for numerous medical conditions to improve their health. One such condition is multiple sclerosis (MS), an autoimmune disorder characterized by demyelination of axons in the brain and spinal cord. Lesions in the brain’s central sudomotor pathway worsen MS symptoms that evoke heat accrual. 1​ ​2​ Termed as Uhthoff’s phenomenon, more adverse impairments occur as increases in core temperature of as little as 0.5°C. 1​ 2​ 3​ 4​ 5​ ​6​ A core temperature of 36.5°C, easily exceeded by MS patients at rest or during exercise, blocked action potential along demyelinated axons to worsen symptoms. 3​ ​4​ ​6​ Elevated core temperatures may impair thermoregulation by reducing cutaneous vasodilation and evaporative heat loss, 1​ ​2​ delaying the start of sweating, 7​ ​8​ or increasing the temperature threshold required to initiate sweating. 9​ ​10​ Endurance training for 15 weeks by MS patients did not improve evaporative heat loss. 11​ Heat intolerance and Uhthoff’s phenomenon result from limited evaporation during exercise as MS patients incur heat strain. 1​ 2​ 3​ ​4​

Prescribing exercise to MS patients is a catch-22; while it maintains or restores neurological health, its heat production increases core temperatures that exacerbate body heat accrual. 1​ 3​​ ​4​ During exercise, MS patients must limit heat accrual that inevitably increases core temperatures and fatigue. 6​ ​7​ Reliance on other heat transfer mechanisms may address this issue. Evaporation removes up to 85% of accrued body heat during exercise, yet conduction is an underutilized mechanism that sees objects of different temperatures transfer heat upon contact. Once identified by Aristotle, heat transfer is based on the objects’ temperature gradient that became known as the Mpemba effect. 12​ ​13​ Evidence also shows that dermal and skeletal muscle vasculature intrinsically restore thermal balance in response to exercise-induced heat accrual. 5​ Regional blood flows and cutaneous insulation determine which body parts are best for conduction. 14​ Those with high blood flows and large surface/volume ratios, such as the palm, which connect to its dermal layer’s reticular veins, also known as retia venosa, rapidly transfer heat to the skin surface. 14​ 15​ ​16​ Palms also have high thermal conductivity and low skin thickness to hasten heat transfer. 17​

As a result, PC, whereby cold objects contact the hand’s volar surface, is used for conductive heat transfer. 14​ PC aids heat removal, 18​ exercise performance, 19​ and physiology 20​ ​21​ for healthy subjects in thermoneutral environments. The magnitude of benefit appears to be based on the type of exercise, subject, and PC stimulus examined. 14​ 18​​ 19​ 20​ ​21​ While vasomotor physiology dictates the blood volume the palm receives, high core temperatures see that it receive up to 10-fold more blood than non-glabrous body parts. 14​ ​22​ Only one study assessed PC’s impact on MS patients; they did two thermoneutral (22°C) walks with, or without, the treatment. 15​ Results included a significant ergogenic effect, as PC saw them walk 35% greater distance. Yet, thermal data were not collected, which limit the understanding of how PC helps MS patients and is a knowledge gap in the literature. 15​ To address this gap, the current study examines PC on heart rates (HRs) and thermal outcomes before, during and after exercise by MS patients. It was hypothesized that intermittent PC would reduce HRs and enhance conductive heat transfer (thermal flux [TFX]) to improve thermoregulation, compared with a no-cooling control condition. If the hypothesis is affirmed, PC may aid MS patients during exercise by hastening heat transfer rates from their bodies.

## Methods

### Study participants

Seven women and two men (mean±standard error of the mean; age 46.4±3.8 y and mass 75.1±3.8 kg) with relapsing remitting MS, characterized by cyclic periodic returns of symptoms throughout their lives with occasional increases in severity, gave informed written consent to participate. The sample, for which an a priori power analysis was not conducted, has a similar size to the only other study to assess PC in MS patients. 15​ However, a post hoc power analysis also revealed that a minimum sample of 10 subjects was required; 23​ since the current sample fell short of that requirement, it serves as a pilot study. Study procedures were approved by a university-based IRB (# 23.0333). To perform activities of daily living, one subject required a cane to ambulate that would yield a score of 6.0 on the Kurtzke Expanded Disability Scale but was able to perform the current workouts. 15​ All other subjects fell within the range of 2.0–3.5 on that same scale, meaning that, despite mild to moderate disability, they were fully ambulatory.

Inclusion criteria required subjects to be in good health and capable of performing moderate intensity exercise. Exclusion criteria required that they should not have the following cardiopulmonary or metabolic conditions: diabetes, asthma, hypertension, heart disease, resting tachycardia, irregular heartbeat, hyperthyroidism, or convulsive disorders. Their prescribed medication use included drugs to modify the disease’s spread in their bodies, such as Ocrevus and Lemtrada. In addition, since subjects had relapsing remitting MS, some used medications such as prednisone to mitigate acute relapses of their disease. To begin laboratory visits, they were encouraged to drink water ad libitum, as study procedures would not allow them to ingest fluids once data collection began. The range of laboratory temperature and humidity values for data collection was 22–23°C and 39–43%, respectively. All study procedures complied with this journal’s ethical guidelines. 24​

### First laboratory visits

**Fig. 1:**
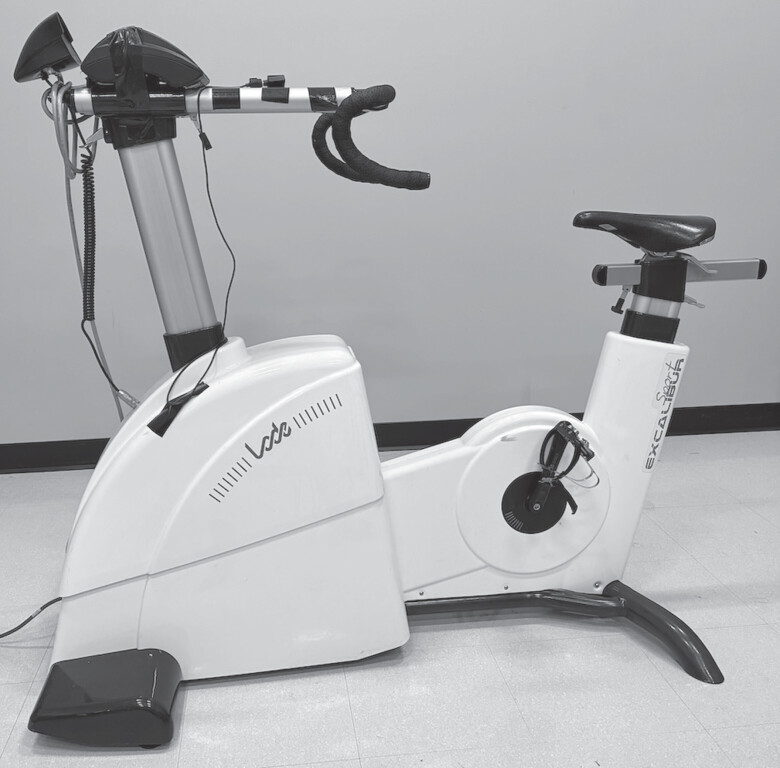
The current study cycle ergometer (LODE Sport Excalibur; Groningen, Netherlands).

Each MS patient made three laboratory visits separated by at least 7 days to limit the likelihood of a training effect. First visits lasted 20–30 minutes and included anthropometric data collection and VO _2_ max estimation. An Astrand–Rhyming test using a stationary cycle ergometer (Excalibur Sport; Lode BV, Groningen Netherlands) estimated aerobic capacity. The workloads achieved during the Astrand–Rhyming test also identified pedal resistance for workouts on their subsequent visits. To begin this test, they pedaled at 50 rpm against 0 kg of added resistance. As they continued at 50 rpm, the resistance was raised based on their gender and estimated fitness level. HR measurements occurred at the end of each minute for 6 consecutive minutes of the test. The HR was anticipated to plateau (less than five beats/minute increases over consecutive minutes), and fall within a 125–170 bpm range, by the end of the 5th and 6th minutes. If those criteria were met, the test was concluded. If not, the resistance was adjusted and the test repeated for another 6-minute period until the said criteria were met. Once the test was completed, their first visit was done. The absolute and relative estimated VO _2_ max values were 2.48±0.34 L min ^−1^ and 32.98±3.97 ml kg ^−1^ min ^−1^ , respectively. An ergometer image is shown in Fig. 1 .

### Second and third laboratory visits

**Fig. 2:**
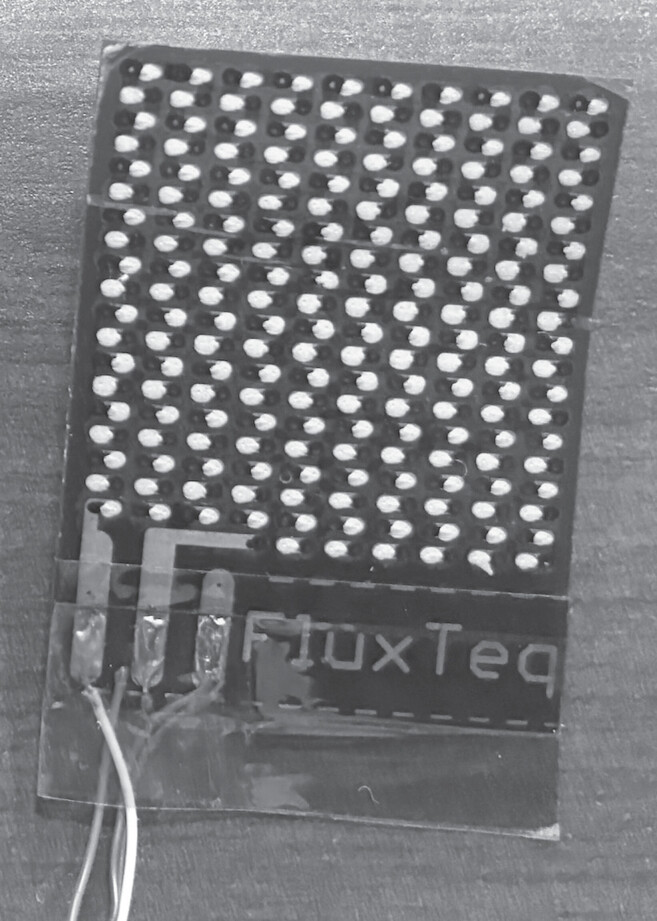
A dermal sensor (FluxTeq; Blacksburg, VA).

The final two visits saw MS patients do identical cycling workouts on the same ergometer used on their first visit. Workouts occurred with and without palm cooling (PC and noPC) whose sequence was decided by a coin flip to begin second visits. Each workout’s purpose was body heat accrual, so that data were compared with and without PC to test the hypothesis. To begin second and third visits, subjects sat for 10 minutes as they were prepped for data collection. The HR was recorded with a telemetry-based watch (Polar; Kempele, Finland). Other dependent variables included tympanic temperature (TT) measured using a hand-held device (Braun; Winamac IN) to estimate the core temperature. 25​ 26​ ​27​ We chose the hand-held device since esophageal or rectal measurements entail probe insertion, and disposable telemetry pills are also a logistical challenge for exercise studies. 17​ The literature claims that body sites to estimate core temperatures should have high thermal conductivity and thin tissue thickness. 17​ Thus, the TT was chosen to estimate the core temperature. A dermal sensor (FluxTeq; Blacksburg, VA) was taped to the monitored palm temperature (PT) and its TFX; the latter measures heat transfer per unit surface area. A dermal sensor image is shown in Fig. 2 .

**Fig. 3:**
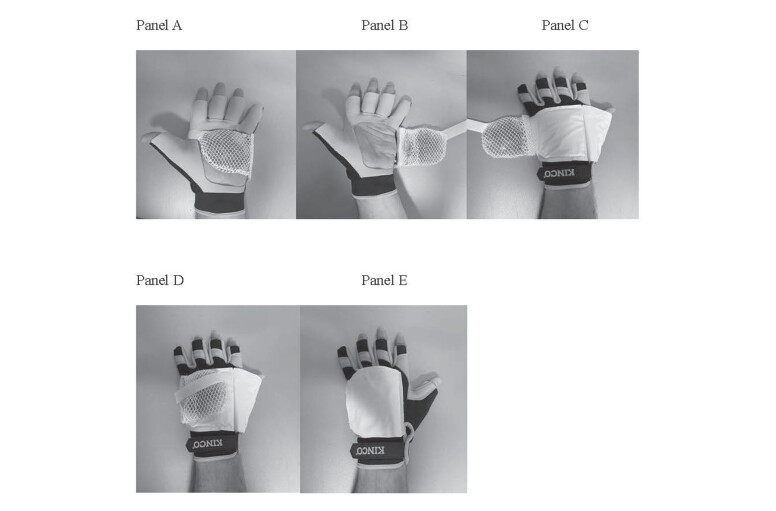
Glove front (Panels A and B) and back (Panels C–E) images. They have a movable flap to hold a 7.6 cm gel pack. The back’s insulated compartment may temporarily store a gel pack for later use. The flap adheres to the front (for PC) or back (for temporary storage) with Velcro.

The sensor had a 6.45 cm ^2^ area, a 380 µm thickness, and a TFX range of±150 kW m ^−2^ . Prior research has identified the relationships between skin temperature, TFX and core temperature. 17​ Sensors were calibrated individually by manufacturers. Each sensor had its own specification sheet provided to researchers. Data acquisition software, used concurrently with the dermal sensors, provided an entry for room temperature values that were incorporated into each TFX measurement. They had a heat flux sensor sensitivity range of 1.38–1.40 mV/W/m ^2^ , and a heat flux measurement accuracy of±0.03 mV/W/m ^2^ or±2%. The dermal sensors included a type T thermocouple to measure temperatures. The sensor was interfaced with a computer to obtain data in real time at 60 Hz. Patients then donned the ( Fig. 3 ) gloves, which were worn without disrupting the sensor’s operation.

**Fig. 4:**
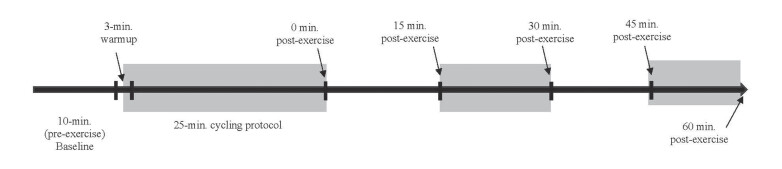
The PC workout timeline, with the times of the PC treatment’s administration.

Gloves were worn on each hand for both workouts. For PC workouts, pre-cooled gel packs (10.6°C) were inserted into, and removed from, the glove’s pouch at specific times for intermittent cooling. Gel packs remained refrigerated at 10.6°C until used. When applied against palms, their initial temperature was 10.6°C but was warmed by conduction commensurate with the volume of heat delivered to subject’s hands. Intermittent PC is the best version of the treatment for heat transfer. 19​ 21​​ ​27​ For PC workouts, gel packs were inserted into gloves halfway through the 3-minute warm-up and removed at the end of the 25-minute cycling protocol. New gel packs were inserted 15 minutes post-exercise, removed 30 minutes post-exercise, and new gel packs were again reinserted 45 minutes post-exercise and remained until the 60-minute recovery period concluded. Fig. 4 shows the PC workout timeline, with the times of the treatment’s administration. Yet, for noPC workouts, gel packs were not used at all.

For the second and third visits, after the 10-minute pre-exercise period concluded, the following dependent variables were collected: HR, TT, PT, and TFX. Workouts began as patients did a 3-minute warm-up on the ergometer by pedaling against 0 kg resistance at 50 rpm. After the warm-up, they immediately continued to pedal at 50 rpm for 15 minutes against a resistance of 65% of their peak Astrand–Rhyming workload. After 15 minutes, the resistance was reduced to 45% of their peak Astrand-Rhyming workload as they immediately continued to pedal for 7 minutes at 50 rpm. After the 7-minute period, they sat for 60 minutes. Workouts entailed identical 25-minute cycling protocols. HR, TT, PT, and TFX were also collected at the end of warm-ups, the 5th and 15th minutes of exercise, and the 30th, 45th, and 60th minutes of recovery. Second and third laboratory visits lasted 95 minutes each.

### Statistics

**Fig. 5:**
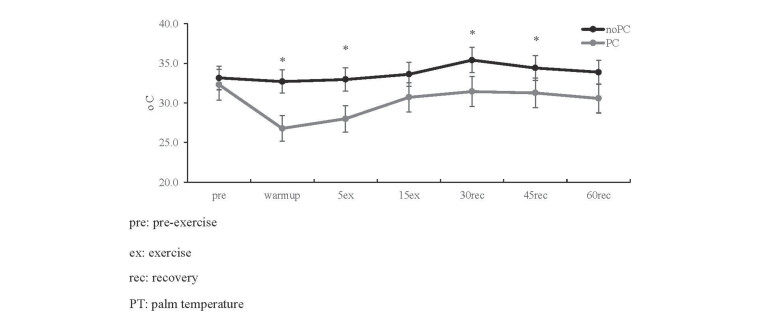
PT results (mean±95% confidence intervals). Asterisks denote sources of the significant differences.

Using a within-subjects design, second and third visit data were compared. HR, TT, PT, and TFX were first examined for outliers with *Z* -scores. Data that provided *Z* -scores of≥±1.96 were eliminated from further analyses. Next, compliance to analysis of variance (ANOVA) assumptions (normality, equal variances, and independence) was assessed. According to the dependent variable, data were compared with two-factor (treatment and time) ANOVAs, with repeated measures per independent variable. To limit type I error rates from the high number of inter-time comparisons per dependent variable analysis, a Bonferroni correction was made. For the current study, a standard *α* value (0.05) was divided by the number of possible temporal comparisons (21) for dependent variables with seven time measurements to yield a Bonferroni-adjusted *α* value of 0.0024 per ANOVA computation. Partial eta squared ( *η* ^2^_*p*_ ) values were provided for ANOVA results that yielded significant inter-treatment differences. Paired *t* -tests, to locate sources of significant differences, served as the post-hoc. In addition, Cohen’s *d* for paired comparisons was provided where significant inter-treatment differences occurred, and 95% confidence intervals per dependent variable are shown in **Figs.** 5 **–** 8 . Since prior MS thermal and HR data from PC do not exist, to better understand current results, inter-workout percentage differences at significant time points for dependent variables with two-way interactions are provided. Pearson’s Product Moment Correlations assessed relationships between TFX and PT, as well as HR and TT, per workout (PC and noPC). Correlations were significant at *α* < 0.05.

**Fig. 6:**
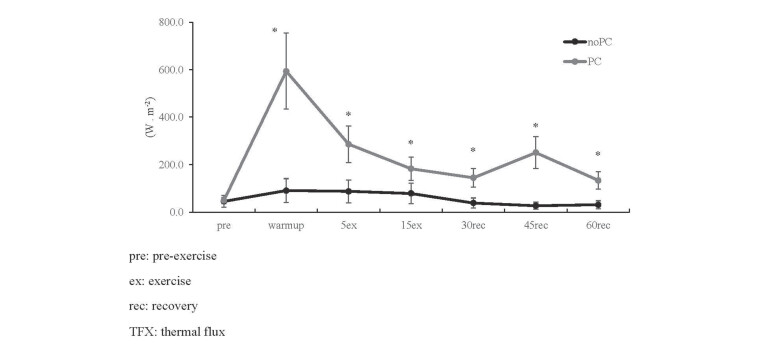
TFX results (mean±95% confidence intervals). Asterisks denote sources of the significant differences.

**Fig. 7:**
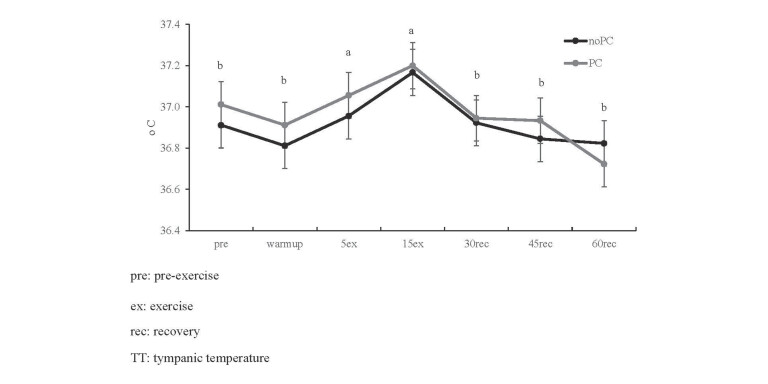
TT results (mean±95% confidence intervals). Letters denote significant ( *a* > *b* ) inter-time differences.

**Fig. 8:**
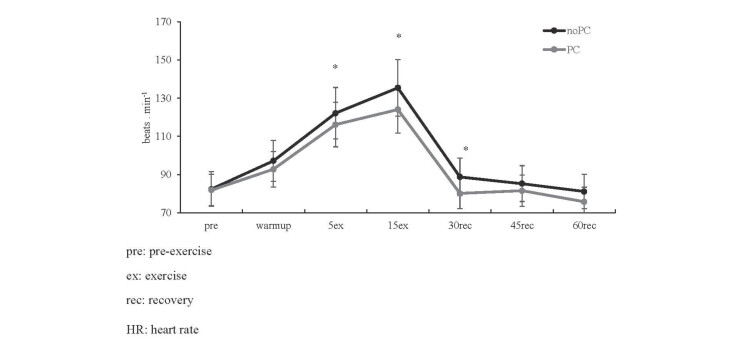
HR results (mean±95% confidence intervals). Asterisks denote sources of the significant differences.

## Results

No MS patients were injured from their pilot study participation. They all completed three visits despite feeling overheated during workouts. *Z* -scores revealed that one patient’s HR data included outliers. The outliers were HR data collected from a single workout. Consultation with their physician confirmed a change in the patient’s medication during their participation that raised HR values for their final workout and created the data outliers. The consultation confirmed *Z* -score results and justified outlier removal. The medication change was not due to their study participation. Only with that data removed were all ANOVA assumptions for HR data met, which then proceeded with an *n* =8. *Z* -score evaluations of the patient’s other dependent variables revealed no outliers; thus, these data were used for statistical analyses. All other pre-exercise dependent variable data were compliant to ANOVA assumptions.

PT results ( Fig. 5 ) had a significant ( *p* =0.00002 and *η* ^2^_*p*_ =0.39) two-way interaction. The post-hoc identified that noPC values were significantly higher than those for PC workouts at the end of warm-ups (Cohen’s *d* =1.5), the 5th minute of exercise (Cohen’s *d* =1.4), and the 30th (Cohen’s *d* =1.13) and 45th (Cohen’s *d* =1.13) minutes of recovery. Intermittent PC elicited (1) higher temperature gradients, (2) lower PT values, and (3) the two-way interaction. Inter-workout PT percentage differences across significant time points were 18.0% (end of warm-ups), 15.2% (5th minute of exercise), 11.3% (30th minute of recovery), and 9% (45th minute of recovery). Fig. 5 plots PT results per treatment over time.

TFX results ( Fig. 6 ) also had a significant ( *p* =0.0004 and *η* ^2^_*p*_ =0.39) two-way interaction. Post-hoc analysis showed that PC values were significantly higher than those for the noPC treatment at the end of warm-ups (Cohen’s *d* =1.6), the 5th (Cohen’s *d* =1.5) and 15th (Cohen’s *d* =1.2) minutes of exercise, and the 30th (Cohen’s *d* =1.5), 45th (Cohen’s *d* =1.0), and 60th (Cohen’s *d* =1.4) minutes of recovery. Inter-workout percentage differences across significant TFX time points were 552% (end of warm-ups), 225% (5th minute of exercise), 132% (15th minute of exercise), 275% (30th minute of recovery), 829% (45th minute of recovery), and 329% (60th minute of recovery). With TFX measured as W m ^−2^ , absolute inter-treatment differences for the significant time points were 502.8 (end of warm-up), 197.9 and 104.1 (5th and 15th minutes of exercise, respectively), and 106.3, 223.8, and 108.8 (30th, 45th, and 60th minutes of recovery, respectively). Fig. 6 plots the TFX results per treatment over time.

TT results ( Fig. 7 ) had a significant ( *p* =0.0008) time main effect. Post-hoc analysis showed the 5th and 15th minutes of exercise had significantly higher TT values than the other time points. Fig. 7 plots the TT results per treatment over time. HR results appear in Fig. 8 . Its data analysis yielded a significant ( *p* =0.00003 and *η* ^2^_*p*_ =0.22) two-way interaction. The post-hoc showed that noPC values were significantly higher than those for PC at the 5th (Cohen’s *d* =0.33) and 15th (Cohen’s *d* =0.52) minutes of exercise, and the 30th (Cohen’s *d* =0.6) minute of recovery. Inter-workout percentage HR differences for the significant time points were 4.9% (5th minute of exercise), 8.4% (15th minute of exercise), and 9.8% (30th minute of recovery). Such changes from PC denote less physiological strain, perhaps from the improved HR recovery, autonomic balance, or higher stroke volume. Fig. 8 plots the HR results per treatment over time.

**Fig. 9:**
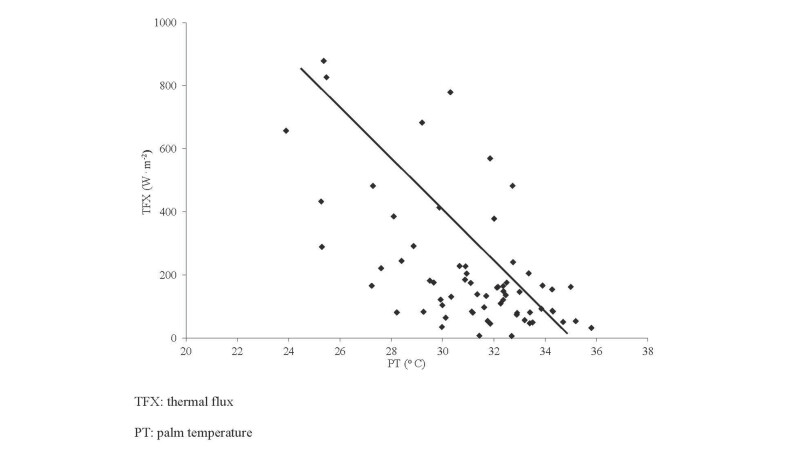
The TFX-PT data plot from PC workouts.

**Fig. 10:**
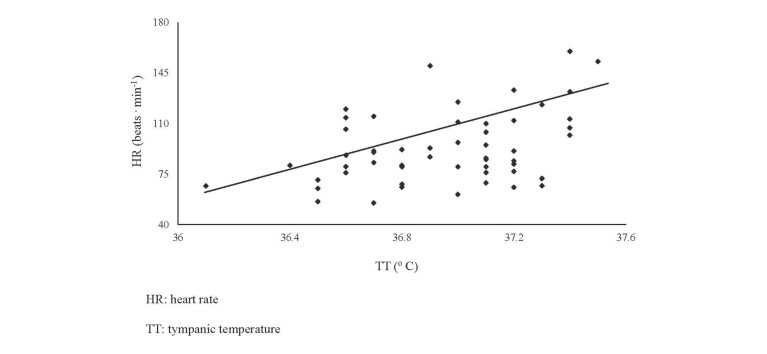
The HR-TT data plot from PC workouts.

Pearson's Product Moment Correlation results show a non-significant relationship between TFX and PT for noPC workout data. Yet, the PC treatment produced a significant ( *r* =− 0.6; *r* ^2^ =0.35) and negative TFX-PT correlation. Fig. 9 shows the TFX-PT data plot for PC workouts. In a similar fashion, the HR-TT data relationship from the noPC workout data was not significant. Yet, the HR–TT relationship with PC workout data had a significant positive correlation ( *r* =0.41; *r* ^2^ =0.17). Fig. 10 displays the HR–TT data plot for PC workouts. Since *r* ^2^ >15% are typically meaningful in clinical research, 28​ **Figs.** 9 **and** 10 imply that the variance in TFX and HR may in part be accounted for by temperature measurements obtained from PC workouts.

## Discussion

The four current dependent variables exhibited either a two-way interaction or main effect despite a lowered α provided by the Bonferroni adjustment. This speaks to the possible physiological merits of intermittent PC in MS patients. While evaporation removes up to 85% of excess heat during exercise, it was hypothesized that it affords MS patients less heat transfer. 29​ Due to their limited evaporation, MS patients may derive comparatively more benefit from conduction when they use PC 16​ 29​​ 30​ 31​ ​32​ ( Fig. 6 ). TFX results from PC are high for exercise done under thermoneutral conditions without insulative clothing. 14​ ​30​ Greater TFX occurs as large temperature gradients are created and maintained, as they enable more heat transfer. 19​ ​21​ Compared to noPC, PC also saw significantly lower HRs during and after workouts. It is important to explain how current HR and TFX results compare to other studies.

Using randomized within-subject designs, PC studies with healthy volunteers performing intense exercise saw significant ergogenic, thermal, and physiological benefits as compared to noPC treatments. 19​ ​20​ ​33​ The ergogenic effects were accompanied by significant inter-treatment heat transfer, 19​ ​33​ HR, 20​ and lactate clearance 19​ ​20​ benefits. Each study entailed repetitive bouts of intense exercise, with larger inter-treatment ergogenic effects over subsequent bouts as presumably more body heat accrued. 19​ ​20​ ​33​ Within-subject designs that assessed moderate intensity efforts by healthy persons, not unlike the current exercise protocol, also showed PC’s benefits. 14​ 18​​ ​34​ Under thermoneutral conditions, PC aided recovery from 30 minutes of walking on a 15% incline at 4 km h ^−1^ . 34​ Post-exercise PC improved recovery for most variables and had significant inter-treatment benefits as compared to a noPC treatment. 34​

In an investigation that quantified heat transfer with the current study’s dermal sensors, PC was assessed during submaximal stationary cycling against prescribed workloads. 18​ Healthy subjects ( *n* =31) did two workouts: one with and one without continuous PC. 18​ There was significantly lower TT, and significantly more TFX, values from continuous PC. Greater heat transfer from PC likely reduced TT values. 18​ Differences in TT results for the current and former 18​ studies may be due to the subjects examined, pre-exercise TT values, and the recovery period duration in which data were collected. Unlike MS patients, healthy subjects could likely rely on both evaporation and PC-induced conduction to lower their TT. 18​ In addition, healthy subjects had a mean pre-exercise TT of 36.11°C, while for current MS patients this measurement was 36.96°C. 18​ Finally, the nature of their condition may require MS patients more post-exercise recovery time, as they still receive an intermittent PC treatment, to significantly lower their TT values. This helps explain lower TT values from PC in the former, but not the current, study. 18​ PC’s effects were also assessed in healthy subjects as they exercised in insulated clothing. 14​ They did identical treadmill walks until a 39°C esophageal temperature was reached; then, a PC or noPC treatment was administered as they recovered. 14​ Post-exercise recovery with PC had a faster heat loss rate (0.8±0.3°C h ^−1^ ) than noPC (0.4±0.2°C h ^−1^ ). 14​ Exercise intensity need not be high to derive PC benefits; current results concur with prior studies that saw also better heat transfer and lower HRs. 14​ 18​​ 19​ ​20​ 33​​ ​34​ A prior study examined PC in MS patients. 15​ Unlike the current project, the prior study saw higher HRs from PC that was attributed to the longer walking distance covered when the treatment was used. 15​ In contrast to the study by Grahn et al., the current study did not measure ergogenic effects but rather had MS patients do identical workouts against prescribed workloads. 15​ Unlike the study by Grahn et al., current HR results agree with findings from a rowing ergometry study with three workouts, two of which entailed intermittent PC. 15​ ​20​ With a noPC workout as the third, there was a significant inter-treatment (noPC>PC) effect for the HR. 20​ With lower HRs from PC workouts, subjects also covered a greater distance rowed. 20​ Significance in the rowing ergometry study was attributed to vagotonia derived from cutaneous cold application that raised parasympathetic activity and lowered HRs. 20​ Given higher core temperatures with MS, it is possible, although only speculative, that PC’s cold application had an even bigger vagotonia response that led to a sharper HR decline and greater inter-treatment HR differences in the current study.

Like HR data, current TFX results had a significant two-way interaction, with more heat transfer, as measured by the dermal sensors, from PC. It is also important to compare TFX outcomes to those from other studies. With two workouts composed of 25 minutes of steady-state cycle ergometry followed by 30 minutes of recovery, 31 healthy subjects did each workout either with, or without, continuous PC. 18​ Inter-workout TFX differences were significant and 32% higher from continuous PC. 18​ A within-subject design compared TFX values to assess intermittent PC’s impact across two workouts, each composed of three 20-second cycling sprints separated by 2 minutes of active recovery. 33​ With workouts composed solely of either a PC or noPC treatment, there was a significant two-way interaction for TFX. The largest inter-workout difference was observed for PC, yielding a 386% higher value than the noPC treatment. 33​ Presumably, inter-study intensity (moderate vs. supramaximal), subject (healthy vs. MS), and PC application (continuous vs. intermittent) disparities account for TFX differences among the current and prior studies. 18​ ​33​ , Fig. 5 shows the inter-treatment differences that illustrate the heat accrual problem in MS patients, and how conduction from intermittent PC may help to abate this problem.

Intermittent, unlike continuous, PC leads to larger temperature gradients that hasten heat transfer. 18​ ​31​ Large gradients may aid MS patients with limited evaporation. 1​ ​2​ 21​​ 35​​ ​36​ This heat transfer phenomenon is called cold-induced vasodilation (CIVD) and may in theory explain current results. While cold application typically elicits vasoconstriction, CIVD has the opposite effect if (1) core temperatures are high and (2) enough norepinephrine binds to adrenergic receptors that control the hand’s blood distribution. 19​ 37​​ 38​ 39​ 40​ ​41​ As core temperatures increase, there is up to a 500% rise (up to 8 L min ^−1^ ) in blood flow to hands during CIVD. 19​ 21​​ 42​​ ​43​ ​45​ Exercise creates both high core temperatures and observed norepinephrine binding to adrenergic receptors. 46​ 47​ ​48​ While vasomotor physiology regulates blood flow to retia venosa, the palm’s surface temperature may fluctuate by up to 10°C during CIVD. 21​ 36​​ ​46​ Yet, it is important to note that CIVD was not measured in the current study, so its impact on MS patients is speculative.

At an air temperature of 27°C, the palm dissipates 150–220 W m ^−2^ of heat. 46​ While PC elicits higher TFX values with exercise, 18​ ​33​ current MS patient results exceed those seen previously. 46​ Yet, since current TFX values are a function of large temperature gradients from intermittent PC, it may be erroneous to assume even colder stimuli applied to the palm would yield a larger magnitude of CIVD and more heat transfer. 20​ A rowing study noted, despite a significant ergogenic effect from intermittent PC, it offered no additional heat transfer compared to a noPC treatment. 20​ Results implied that the gel pack temperature (8.1°C) was too low to cause CIVD. 20​ This statement is supported by research that saw hand immersion at 8°C during exercise impaired muscle contractility, heightened fatigue and evoked no CIVD response. 49​ 50​ ​51​ Acclimatization to immersive hand exercise at 8°C for 2–3 weeks did not improve the likelihood of CIVD. 49​ 50​ ​51​ Subsequent research implied that PC at 10–10.6°C may allow the largest thermal gradient without sustained hand vasoconstriction and is similar to the initial temperature of the current gel packs. 37​ ​52​ Yet, papers that implied colder stimuli are counterproductive to CIVD did not assess MS patients.

### Limitations and conclusions

As was done with healthy subjects, future research should identify an optimal PC temperature for MS patients during exercise. 37​ ​52​ Future research should also validate PC’s use in this population. Potential future study limitations include differing disability levels among MS patients. Current study limitations also include TT to estimate core temperatures. Exercise adherence and compliance for disabled subjects is easier when the TT is assessed using a hand-held device, particularly when more invasive methods may compromise their exercise performance, yet it is important to note that esophageal and rectal measurements are established as true representations of core body temperatures. 17​ Other current limitations include the sample size ( *n* =9), the absence of sweat rate and exercise performance data, and a focus on the subtype (relapsing remitting) of MS.

Since most MS patients may rely on conduction to a greater extent than healthy persons for heat transfer, PC appears promising to mitigate heat intolerance. Unlike other options, PC is cheap, simple, and safe to administer. 53​ Given this background and the current PC results in an MS pilot study, the feasibility of future investigations, including clinical trials, appear warranted.

## References

[1] Panginikkod S Rayi A Rocha Cabrero F Uhthoff Phenomenon StatPearls 2022 29261916

[2] Razi O Tartibian B Teixeira A M Thermal dysregulation in patients with multiple sclerosis during SARS-CoV-2 infection. The potential therapeutic role of exercise Mult Scler Relat Dis 2022 59 103557 10.1016/j.msard.2022.103557 PMC8785368 35092946

[3] Davis S L Wilson T E White A T Thermoregulation in multiple sclerosis J Appl Physiol 2010 109 1531 1537 20671034 10.1152/japplphysiol.00460.2010 PMC2980380

[4] Jain A Rosso M Santoro J D Wilhelm uhthoff and uhthoff’s phenomenon Mult Scler 2020 26 1790 1796 31621479 10.1177/1352458519881950

[5] Langan S P Casa D J Kwon O S Thermosensitivity of the microvasculature: Molecular and physiological mechanisms in skeletal muscle-a narrative review J Therm Biol 2025 134 104331 41240785 10.1016/j.jtherbio.2025.104331

[6] Rasminsky M The effects of temperature on conduction in demyelinated single nerve fibers Arch Neurol 1973 28 287 292 4696011 10.1001/archneur.1973.00490230023001

[7] Krupp L B McLinskey N MacAllister W S Fatigue in multiple sclerosisIn:Multiple Sclerosis Therapeutics CRC Press 2007 805 818

[8] Petajan J H White A T Recommendations for physical activity in patients with multiple sclerosis Sports Med 1999 27 03 179 191 10222541 10.2165/00007256-199927030-00004

[9] Huang M Jay O Davis S L Autonomic dysfunction in multiple sclerosis: Implications for exercise Auton Neurosci 2015 188 82 85 25458432 10.1016/j.autneu.2014.10.017 PMC4852554

[10] Racosta J M Kimpinski K Morrow S A Autonomic dysfunction in multiple sclerosis Auton Neurosci 2015 193 1 6 26070809 10.1016/j.autneu.2015.06.001

[11] Davis S L Wilson T E Vener J M Pilocarpine-induced sweat gland function in individuals with multiple sclerosis J Appl Physiol 2005 98 05 1740 1744 15640392 10.1152/japplphysiol.00860.2004

[12] Aristotle in Webster E W Meterologica I Oxford Oxford University Press 1923 348b 349a

[13] Tao Y Zou W Jia J Different ways of hydrogen bonding to water-why does warm water freeze faster than cold water? J Chem Theory Comput 2017 13 01 55 76 27996255 10.1021/acs.jctc.6b00735

[14] Grahn D A Dillon J L Heller H C Heat loss through the glabrous skin surfaces of heavily insulated, heat-stressed individuals J Biomed Eng 2009 131 171005 10.1115/1.3156812 19640130

[15] Grahn D A Murray L S. Heller H C Cooling via one hand improves physical performance in heat sensitive individuals with multiple sclerosis: A preliminary study BMC Neurol 2008 8 14 18474113 10.1186/1471-2377-8-14 PMC2396661

[16] Walloe L Arterio-venous anastomoses in the human skin and their role in temperature control Temperature 2016 3 01 92 103 10.1080/23328940.2015.1088502 PMC4861183 27227081

[17] Xu X Karis A J Buller M J Relationship between core temperature, skin temperature, and heat flux during exercise in heat Eur J Appl Physiol 2013 113 2381 2389 23775374 10.1007/s00421-013-2674-z

[18] Patel N L Quesada P M Wellwood J Continuous palm cooling’s effect on heat transfer and physiology Isok Exerc Sci 2024 32 145 153

[19] Caruso J F Barbosa A G Erickson L Intermittent palm cooling’s impact on resistive exercise performance Int J Sports Med 2015 36 814 821 26038879 10.1055/s-0035-1547264

[20] OBrien I T Kozerski A E Gray W D Use of gloves to examine intermittent palm cooling’s impact on rowing ergometry workouts J Strength Cond Res 2021 35 931 940 33629973 10.1519/JSC.0000000000003561

[21] Kwon Y S Robergs R A Schneider S M Effect of local cooling on short-term, intense exercise J Strength Cond Res 2013 27 2046 2054 23085975 10.1519/JSC.0b013e3182773259

[22] Adams W M Hosokawa Y Adams E L Reductions in body temperature using hand cooling versus passive rest after exercise in the heat J Sci Med Sport 2016 19 11 936 940 27012727 10.1016/j.jsams.2016.02.006

[23] https://www.statscalculators.com/calculators/hypothesis-testing/sample-size-and-power-analysis-calculator

[24] Harriss D J Jones C MacSween A Ethical standards in sport and exercise science research: 2022 update Int J Sports Med 2022 43 1065 1070 36495253 10.1055/a-1957-2356

[25] Ajcevic M Stella A B Furlanis G A novel non-invasive thermometer for continuous core body temperature: Comparison with tympanic temperature in an acute stroke clinical setting Sensors 2022 22 4760 35808257 10.3390/s22134760 PMC9269248

[26] Doohan M A Watzek J T King N Does increased core temperature alter cognitive performance during exercise-induced heat strain? A narrative review J Appl Physiol 2023 135 35 52 37141422 10.1152/japplphysiol.00070.2023

[27] Gray W D Jett M Cocco A R Ergogenic and physiological outcomes derived from a novel skin cooling device J Strength Cond Res 2021 35 391 403 33278269 10.1519/JSC.0000000000003864

[28] Gupta A Stead T S Ganti L Determining a meaningful R-squared value in clinical medicine Academic Med Surg 2024 10.62186/001c.125154

[29] Mundel T Thermoregulatory sweating and evaporative heat loss during exercise: Is the whole greater than the sum of its parts? J Physiol 2020 598 13 2535 2536 32406117 10.1113/JP279944

[30] Lissoway J B Lipman G S Grahn D A Novel application of chemical cold packs for treatment of exercise-induced hyperthermia: A randomized controlled trial Wild Environ Med 2015 26 02 173 179 10.1016/j.wem.2014.11.006 25771030

[31] Maguire K F Wydotis M M Patel N L Palm cooling for heat mitigationIn75th International Astronautical Congress Proceedings Milan, Italy IAC-24,A1,4,x81280 2024

[32] Maguire K F Wydotis M M Vasudevan K Multiple Sclerosis: Challenges to health and exercise performance Am Col Sports Med Health Fit J 2024 28 06 47 50

[33] Wydotis M M Maguire K F Quesada P M Palm cooling’s impact on ergogenic, thermal, and physiological outcomes from high-intensity interval training Isok Exerc Sci 2026 34 154 163 10.1177/09593020261419923

[34] Park J Kim J Effects of cooling glove on the human body’s recovery after exercise and improvement of exercise ability Tech Health Care 2023 31 S259 S269 10.3233/THC-236022 PMC10200210 37066927

[35] Heller H C Grahn D A Enhancing thermal exchange in humans and practical applications Disrup Sci Tech 2012 1 01 11 19

[36] Hsu A R Hagobian T A Jacobs K A Effects of heat removal through the hand on metabolism and performance during cycling exercise in the heat Can J Appl Physiol 2005 30 01 87 104 15855685 10.1139/h05-107

[37] Grahn D A Cao V H Nguyen C M Work volume and strength training responses to resistive exercise improve with periodic heat extraction from the palm J Strength Cond Res 2012 26 2558 2569 22076097 10.1519/JSC.0b013e31823f8c1a

[38] Daanen HA M Finger cold-induced vasodilation: A review Eur J Appl Physiol 2003 89 411 426 12712346 10.1007/s00421-003-0818-2

[39] Shephard J T Rusch N J Vanhoutte P M Effect of cold on the blood vessel wall Gen Pharmac 1983 14 61 64 10.1016/0306-3623(83)90064-2 6131011

[40] Tipton M J Allsop A Balmi P J Hand immersion as a method of cooling and rewarming: A short review J Roy Nav Med Serve 1993 79 125 131 8207704

[41] White G E Wells G D Cold-water immersion and other forms of cryotherapy: Physiological changes potentially affecting recovery from high-intensity exercise Extreme Physiol Med 2013 2 26 10.1186/2046-7648-2-26 PMC3766664 24004719

[42] Manelli A Sangiorgi S Ronga M Plexiform vascular structures in the human digit dermal layer: A SEM corrosion casting morphological study Eur J Morphol 2005 424–5173 177 16982473 10.1080/09243860500359885

[43] Nagasaka T Cabanac M Hirata K Control of local heat gain by vasomotor response of the hand J Appl Physical 1987 63 04 1335 1338 10.1152/jappl.1987.63.4.1335 3693165

[44] Rowell L B Human cardiovascular adjustments to exercise and thermal stress Physiol Rev 1974 54 01 75 159 4587247 10.1152/physrev.1974.54.1.75

[45] Sangiorgi S Manelli A Congiu T Microvascularization of the human digit as studied by corrosion casting J Anat 2004 204 123 131 15032919 10.1111/j.1469-7580.2004.00251.x PMC1571248

[46] Cheung S S Responses of the hands and feet to cold exposure Temperature (Austin) 2015 2 01 105 120 27227009 10.1080/23328940.2015.1008890 PMC4843861

[47] Romanovsky A A Skin temperature: Its role in thermoregulation Acta Physiologica 2014 210 498 507 24716231 10.1111/apha.12231 PMC4159593

[48] Thorsson O Lilja B Ahlgren L The effects of local cold application on intramuscular blood flow at rest and after running Med Sci Sports Exerc 1985 17 06 710 713 4079745 10.1249/00005768-198512000-00016

[49] Geurts C L Sleivert G G Cheung S S Local cold acclimatization of the hand impairs thermal responses of the finger without improving hand neuromuscular function Acta Physiol Scand 2005 183 117 124 15654925 10.1111/j.1365-201X.2004.01374.x

[50] Geurts C L Slivers G G Cheung S S Central and peripheral factors in thermal, neuromuscular, and perceptual adaptation of the hand to repeated cold exposures Appl Physiol Nutr Metab 2006 31 110 117 16604128 10.1139/h05-007

[51] Geurts C L Sleivert G G Cheung S S Local cold acclimatization during exercise and its effect on neuromuscular function of the hand Appl Physiol Nutr Metab 2006 31 717 725 17213886 10.1139/h06-076

[52] Soltysiak S R Colborn C E Dichiara E J Palm cooling temperatures and their comparative impact on human thermal, physiological, perceptual, and ergogenic indices J Sport Sci 2022 40 20 2292 2303 10.1080/02640414.2022.2151750 36463544

[53] Iwata R Kawamura T Okabe F Effects of palm cooling on thermoregulatory-related and subjective indicators during exercise in a hot environment J Therm Biol 2024 120 103803 38382413 10.1016/j.jtherbio.2024.103803

